# Stromal tumor-infiltrating lymphocytes and pathologic response to neoadjuvant chemotherapy with the addition of platinum and pembrolizumab in TNBC: a single-center real-world study

**DOI:** 10.1186/s13058-024-01944-0

**Published:** 2024-12-18

**Authors:** Soong June Bae, Jee Hung Kim, Min Ji Kim, Yoonwon Kook, Seung Ho Baek, Jung Hyun Kim, Sohyun Moon, Seung Eun Lee, Joon Jeong, Yoon Jin Cha, Sung Gwe Ahn

**Affiliations:** 1 Department of Surgery, Gangnam Severance Hospital, Yonsei University College of Medicine, Seoul, Republic of Korea; 2 Institute for Breast Cancer Precision Medicine, Gangnam Severance Hospital, Yonsei University College of Medicine, Seoul, Korea; 3 Division of Medical Oncology, Department of Internal Medicine, Gangnam Severance Hospital, Yonsei University College of Medicine, Seoul, Korea; 4 Department of Pathology, Gangnam Severance Hospital, Yonsei University College of Medicine, Seoul, Republic of Korea

## Abstract

**Background:**

Immunochemotherapy with pembrolizumab has been integrated into clinical practice as part of the standard-of-care for non-metastatic triple-negative breast cancer (TNBC) with high risk. We conducted a real-world study in TNBC patients treated with neoadjuvant chemotherapy to compare pathologic complete response (pCR) rates relative to stromal tumor-infiltrating lymphocytes (sTIL) across different regimens: non-carboplatin, carboplatin-, and pembrolizumab-chemotherapy.

**Patients and methods:**

We analyzed a cohort of 450 patients with TNBC who underwent surgery following neoadjuvant chemotherapy between March 2007 and February 2024. Treatment groups included 247 non-carboplatin, 120 carboplatin, and 83 pembrolizumab-chemotherapy recipients. sTIL was evaluated in biopsied samples. Lymphocyte-predominant breast cancer (LPBC) was defined as tumors with high sTIL (≥ 50%).

**Results:**

The pCR rates were 32% in the non-carboplatin-, 57% in the carboplatin-, and 64% in the pembrolizumab-chemotherapy group. Ninety-two patients (20.4%) had LPBC. In LPBC, the pCR rates did not increase with the addition of carboplatin (50.0% in the non-carboplatin and 41.7% in carboplatin) but reached 83.3% with the addition of pembrolizumab and carboplatin. Among the non-LPBC, the pCR rate increased from 26.7 to 61.1% with the addition of carboplatin, but there was no difference in the pCR rate between the carboplatin and pembrolizumab groups (61.1% and 61.2%, respectively).

**Conclusions:**

In LPBC patients, the addition of carboplatin did not result in an elevated pCR rate; however, the addition of pembrolizumab tended to raise the pCR rate. In non-LPBC, the addition of carboplatin significantly increased the pCR rate, while the addition of pembrolizumab did not have the same effect.

**Supplementary Information:**

The online version contains supplementary material available at 10.1186/s13058-024-01944-0.

## Introduction

Since neoadjuvant pembrolizumab combined with chemotherapy has been shown to increase pathologic complete response (pCR) rates [1], and subsequent administration of adjuvant pembrolizumab post-surgery has extended event-free survival (EFS) compared to neoadjuvant chemotherapy alone in patients with early triple-negative breast cancer (TNBC) [2], immunochemotherapy including pembrolizumab has become the standard of care for managing early TNBC patients at high risk.

Prior to the era of immunochemotherapy with pembrolizumab, anthracycline- and taxane-based (A-T) chemotherapy regimens were the preferred treatment approach in TNBC [3]. Furthermore, investigators endeavored to enhance clinical outcomes by incorporating carboplatin into the A-T regimen. Previous trials have commonly shown that the addition of carboplatin to A-T increases the rate of pCR [4–6], although improvements in EFS were inconsistent [6–8]. Despite the controversy surrounding the addition of carboplatin to A-T, carboplatin-containing chemotherapy has been the most preferred polychemotherapy regimen for TNBC with high risk [1].

Personalizing neoadjuvant chemotherapy with or without pembrolizumab remains a significant challenge in early-stage TNBC, largely due to the limited availability of robust biomarkers. While the KEYNOTE-522 trial demonstrated benefits of pembrolizumab in improving pathological complete response (pCR) and event-free survival (EFS) regardless of PD-L1 expression [9, 10], this does not confirm that all patients derived benefit. Given the substantial clinical burden of immunotherapy-related toxicities [11] and the associated financial costs, there is an urgent need to develop reliable biomarkers to tailor treatment regimens. Compelling evidence suggests that high levels of stromal tumor-infiltrating lymphocytes (sTILs) in pre-treatment biopsy samples are strongly correlated with higher pCR rates in TNBC patients undergoing neoadjuvant chemotherapy [12–17]. However, the role of sTILs to inform treatment decisions regarding the addition or omission of pembrolizumab in early TNBC remains inadequately explored.

In this study, we conducted a real-world study in TNBC patients treated with neoadjuvant chemotherapy, comparing pCR rates relative to sTIL levels across different neoadjuvant chemotherapy regimens: (i) non-carboplatin-chemotherapy (ii) carboplatin-chemotherapy, and (iii) pembrolizumab-chemotherapy.

## Patients and methods

### Study population

In this retrospective cohort study, we included 450 non-metastatic TNBC patients treated with neoadjuvant chemotherapy at Gangnam Severance Hospital, Seoul, Republic of Korea. All patients underwent curative surgery after neoadjuvant chemotherapy between March 2007 and February 2024. Chemotherapy regimen was chosen as per local guidelines at the time of diagnosis. We excluded the patients diagnosed with recurrent or metachronous BC. This study included the patients at clinical stage IIIC. Staging was performed according to the 8th edition of the American Joint Committee on Cancer system, and histologic grading was conducted using the Elston-Ellis modification of the Scarff-Bloom-Richardson grade.

TNBC was defined as estrogen receptor (ER)-negative and progesterone receptor (PR)–negative according to the guidelines of the American Society of Clinical Oncology and the College of American Pathologists using immunohistochemistry results of tumor. HER2 negativity was determined by IHC (score 0 or 1+) or by fluorescent silver in situ hybridization for cases with an IHC score of 2+. Clinical stage information was determined through physical examination and imaging studies, including mammography, breast ultrasonography, and breast magnetic resonance imaging. To rule out distant metastasis, abdominal and chest computed tomography scans, and bone scans, with/without PET-CT, were performed. Information regarding ER, PR, HER2 status, histologic grade, and sTILs was collected using biopsied samples before neoadjuvant chemotherapy. Germline BRCA1/2 mutation status was assessed in whole blood samples. Unknown information was treated as missing values. Pathological complete response (pCR) was defined as ypT0/is N0. The study was approved by the Institutional Review Board, adhering to good clinical practice guidelines under the Declaration of Helsinki.

### Chemotherapy regimen

Among the study population, 247 (54.9%) patients received the non-carboplatin-chemotherapy, 120 (26.7%) received the carboplatin-chemotherapy, and 83 (18.4%) received the pembrolizumab-chemotherapy [1].

The non-carboplatin group included anthracycline-cyclophosphamide followed by taxane regimen (AC/T), and concurrent anthracycline plus taxane regimen, or anthracycline alone or taxane alone regimen. The carboplatin-chemotherapy group included patients with anthracycline-cyclophosphamide followed by taxane-carboplatin regimen or taxane-carboplatin followed by anthracycline-cyclophosphamide regimen. Patients in the pembrolizumab-chemotherapy group were all treated with the KN-522 regimen (8 cycles of pembrolizumab 200 mg, given with 12 cycles of 1 week (W) paclitaxel and 4 cycles of 3 W carboplatin, followed by 4 cycles of 3 W doxorubicin and cyclophosphamide. Five patients were unable to complete 8 cycles of chemotherapy due to adverse effects. In this study, patients who received at least one dose of pembrolizumab were included in the pembrolizumab-chemotherapy group for analysis. Our study population was summarized in Supplementary Fig. 1.

### Stromal tumor-infiltrating lymphocytes

sTILs were evaluated in biopsied samples before neoadjuvant chemotherapy based on hematoxylin and eosin-stained slides following the protocol by the TIL international working group [18]. We previously reported that sTILs of biopsied samples were correlated with that of surgical specimens, allowing evaluation in biopsied samples [19]. The average sTIL levels was counted from all available tumor area for each case, and the average percentage score was reported. In this study, lymphocyte-predominant breast cancer (LPBC) was defined as tumors having high sTIL levels (≥ 50%) [20, 21]. Non-LPBC tumors were classified into two groups; (i) low sTIL (≤ 10%) and (ii) intermediate sTILs (11-49%) [13].

### Statistics

Continuous variables were compared using the Mann–Whitney U test and Student’s *t*-test. Kolmogorov–Smirnov tests were used to assess the normal distribution of the continuous variables. Nominal variables were compared using *χ*^2^ or Fisher’s exact tests. Multivariable binary logistic regression analysis was performed to identify predictive factors for pCR. The variables with *p*-value < 0.05 were included in the multivariable model, and the stepwise backward Wald method was used to arrive at the final model. All statistical analyses were performed using SPSS program version 27.0 (SPSS Inc., Chicago, USA) and R software (https://www.r-projet.org; version 3.6.1). A *p*-value < 0.05 was considered statistically significant.

## Results

### Study population

We identified 450 patients with TNBC who underwent neoadjuvant chemotherapy followed by curative surgery. Baseline demographics and tumor characteristics are described in Table 1. Among these patients, 185 (41.4%) were aged ≥ 50 years, and 188 (41.8%) had histologic grade III tumors. Of these, germline BRCA1/2 mutation testing was conducted in 264 patients, with mutations identified in 58 (22.0%) of them. Most of the patients had clinical T2 or T3 (399/450 [88.6%]) or clinically node-positive tumors (367/450 [81.6%]). Evaluation of sTILs in biopsy specimens was conducted in 369 patients (82.0%), revealing a median sTILs level of 20% (interquartile range [IQR], 3–40%).

**Table 1:** Baseline characteristics of patients according to neoadjuvant chemotherapy regimen (*n* = 450)

	Non-carboplatin (*N* = 247)	Carboplatin (*N* = 120)	Pembrolizumab (*N* = 83)	Total (*N* = 450)	*P*
Age, n (%)					0.630
≤ 50	143 (57.9)	75 (62.5)	47 (56.6)	265 (58.9)	
> 50	104 (42.1)	45 (37.5)	36 (43.4)	185 (41.4)	
Germline BRCA1/2 mutation^*^					0.090
No	86 (81.9)	64 (70.3)	56 (82.4)	206 (78.0)	
Yes	19 (18.1)	27 (29.7)	12 (17.6)	58 (22.0)	
Clinical tumor stage					0.156
1	14 (5.7)	7 (5.8)	6 (7.2)	27 (6.0)	
2	163 (66.0)	91 (75.8)	62 (74.7)	316 (70.2)	
3	51 (20.6)	18 (15.0)	14 (16.9)	83 (18.4)	
4	19 (7.7)	4 (3.3)	1 (1.2)	24 (5.3)	
Clinical nodal stage					0.280
0	47 (19.0)	25 (20.8)	11 (13.3)	83 (18.4)	
1	100 (40.5)	51 (42.5)	43 (51.8)	194 (43.1)	
2	87 (35.2)	36 (30.0)	21 (25.3)	144 (32.0)	
3	13 (5.3)	8 (6.7)	8 (9.6)	29 (6.4)	
Clinical Stage, n (%)					0.133
I, IIA	49 (19.8)	24 (20.0)	13 (15.7)	86 (19.1)	
IIB	75 (30.4)	41 (34.2)	37 (44.6)	153 (34.0)	
IIIA	93 (37.7)	43 (35.8)	24 (28.9)	160 (35.6)	
IIIB	17 (6.9)	4 (3.3)	1 (1.2)	22 (4.9)	
IIIC	13 (5.3)	8 (6.7)	8 (9.6)	29 (6.4)	
Histologic grade^*^, n (%)					0.369^†^
1	4 (2.2)	0	1 (1.3)	5 (1.3)	
2	85 (47.0)	53 (46.5)	44 (55.0)	182 (48.5)	
3	92 (50.8)	61 (53.5)	35 (43.8)	188 (48.5)	
sTILs, median (range)	20 (10–60)	20 (3–40)	10 (3–25)	20 (3–40)	< 0.001
sTILs^*^, %					0.009
< 50	120 (68.2)	90 (78.9)	67 (84.8)	277 (75.1)	
≥ 50	56 (31.8)	24 (21.1)	12 (15.2)	92 (24.9)	
Surgery type					0.512
Lumpectomy	137 (55.5)	74 (61.7)	49 (59.0)	260 (57.8)	
Mastectomy	110 (44.5)	46 (38.3)	34 (41.0)	190 (42.2)	

^*^Missing values ^†^*P*-values were obtained with the Fisher’s exact test. sTILs, stromal tumor-infiltrating lymphocytes

Based on the treatment regimen, the patients were divided as follows: non-carboplatin-chemotherapy group (247 patients [54.9%]), carboplatin-chemotherapy group (120 patients [26.7%]), and pembrolizumab-chemotherapy group (83 patients [18.4%]). The prevalence of LPBC (sTILs ≥ 50%) varied across three groups, with higher frequencies observed sequentially in the non-carboplatin (56/247 [31.8%]), carboplatin (24/114 [21.1%]), and pembrolizumab groups (12 /89 [15.2%]). Other clinicopathological factors did not significantly differ among the three groups. In addition, among patients with available sTILs data, baseline features were comparable across treatment regimens (Supplementary Table 1).

### Pathologic complete response rates according to the regimens

**Fig. 1:**
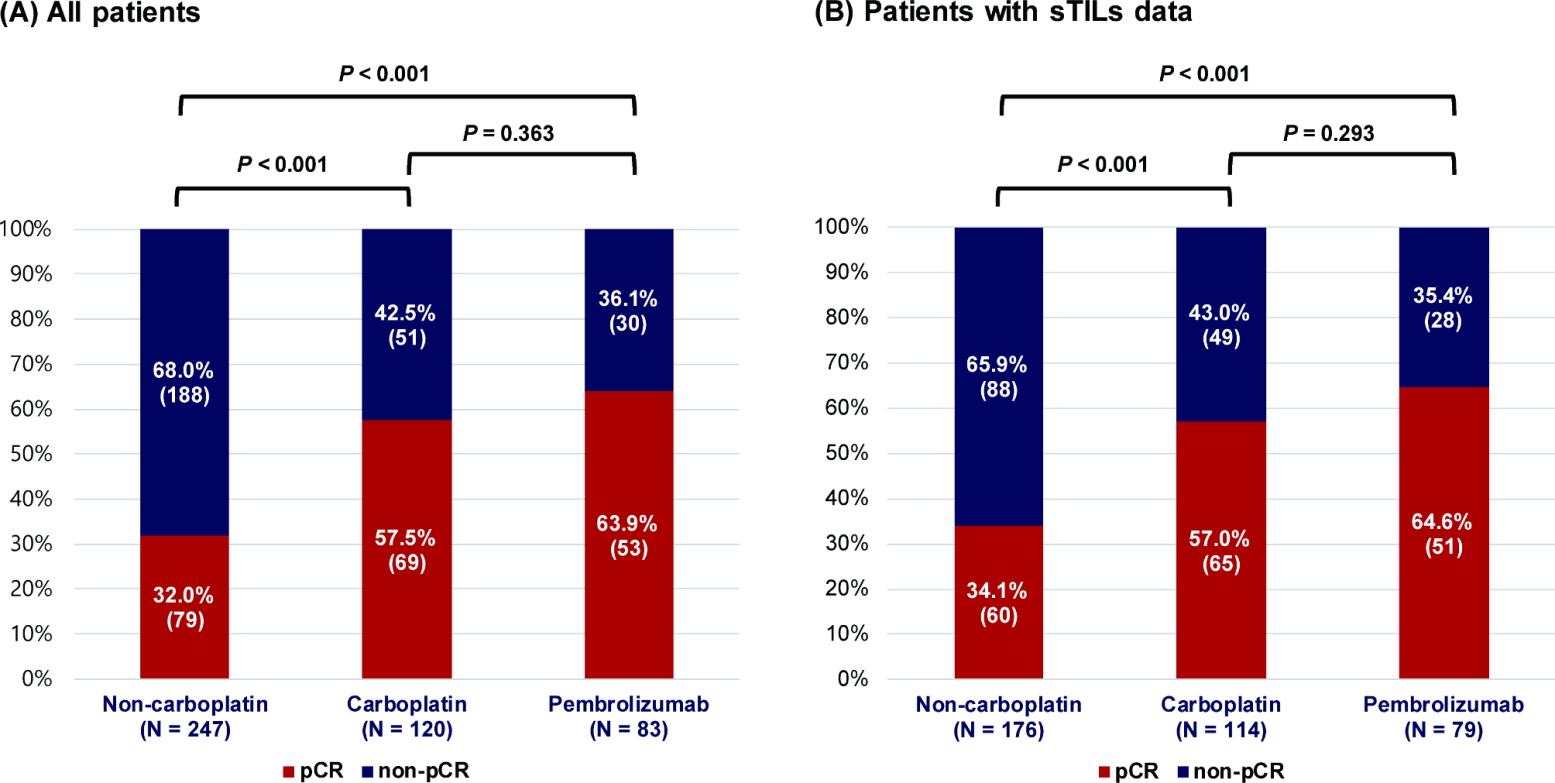
Pathologic complete response (pCR) rate according to neoadjuvant treatment regimens (**A**) in all patients and (**B**) patients with available stromal tumor-infiltrating lymphocytes (sTILs) data

Of 450 patients, 201 (44.7%) achieved pCR after neoadjuvant systemic treatments. Specifically, pCR rates were 32.4% (79/247) in the non-carboplatin chemotherapy group, 57.5% (69/120) in the carboplatin-chemotherapy group, and 63.9% (53/83) in the pembrolizumab-chemotherapy group (Fig. 1A). The pCR rate of the non-carboplatin-chemotherapy group was significantly lower than those of the carboplatin- and pembrolizumab-chemotherapy groups, respectively. However, no statistical difference in pCR rate was noted between the carboplatin- and pembrolizumab-chemotherapy group (*P* = 0.363).

These outcomes of pCR according to the regimens were consistent in the subset of 369 patients evaluated for sTILs: pCR rates were 34.1% (60/176) in the non-carboplatin-chemotherapy group, 57.0% (65/114) in the carboplatin regimen group, and 64.6% (51/79) in the pembrolizumab-chemotherapy group (Fig. 1B).

Multivariable analysis showed that the neoadjuvant chemotherapy regimen was an independent factor for pCR. Compared to the non-carboplatin regimen, the carboplatin (adjusted odds ratio [OR], 2.73; 95% CI, 1.64–4.57; *P* < 0.001) and pembrolizumab (adjusted OR, 4.00; 95% CI, 2.20–7.16; *P* < 0.001) regimens were significantly associated with increased odds of achieving pCR (Table 2). However, in this analysis, high TILs (≥ 50%) was not demonstrated as an independent factor for pCR (Table 2).

**Table 2:** Odds ratio (OR) and 95% confidence interval (CI) for pathologic complete response in 369 patients with available sTILs

	Univariable analysis			Multivariable analysis			
	OR	95% CI	*P*		adjusted OR	95% CI	*P*
Age							
≤ 50	Ref.				Ref.		
> 50	0.69	0.46–1.04	0.079		0.80	0.51–1.26	0.341
Histologic grade			0.074				0.175
1	Ref.				Ref.		
2	3.01	0.33–27.48	0.329		2.17	0.21–22.13	0.512
3	4.55	0.50–41.50	0.179		3.20	0.31–32.78	0.327
Clinical tumor stage			0.126				0.578
1	Ref.				Ref.		
2	0.94	0.41–2.13	0.876		0.83	0.35–1.96	0.666
3	0.62	0.25–1.59	0.322		0.67	0.25–1.78	0.417
4	0.25	0.06–1.13	0.071		0.37	0.08–1.81	0.220
Clinical nodal stage							0.555
0	Ref.				Ref.		
1	1.15	0.66–2.01	0.612		0.90	0.50–1.64	0.730
2	0.78	0.43–1.40	0.399		0.67	0.35–1.28	0.227
3	0.73	0.30–1.77	0.482		0.64	0.241.72	0.379
sTILs							
< 50%	Ref.				Ref.		
≥ 50%	1.27	0.79–2.04	0.322		1.60	0.95–2.69	0.079
Regimen							< 0.001
Non-carboplatin	Ref.				Ref.		
Carboplatin	2.57	1.58–4.16	< 0.001		2.73	1.64–4.53	< 0.001
Pembrolizumab	3.52	2.02–6.14	< 0.001		4.00	2.20–7.16	< 0.001

sTILs, stromal tumor-infiltrating lymphocytes

### Pathologic complete response rates of the regimen groups in relation to sTILs

**Fig. 2:**
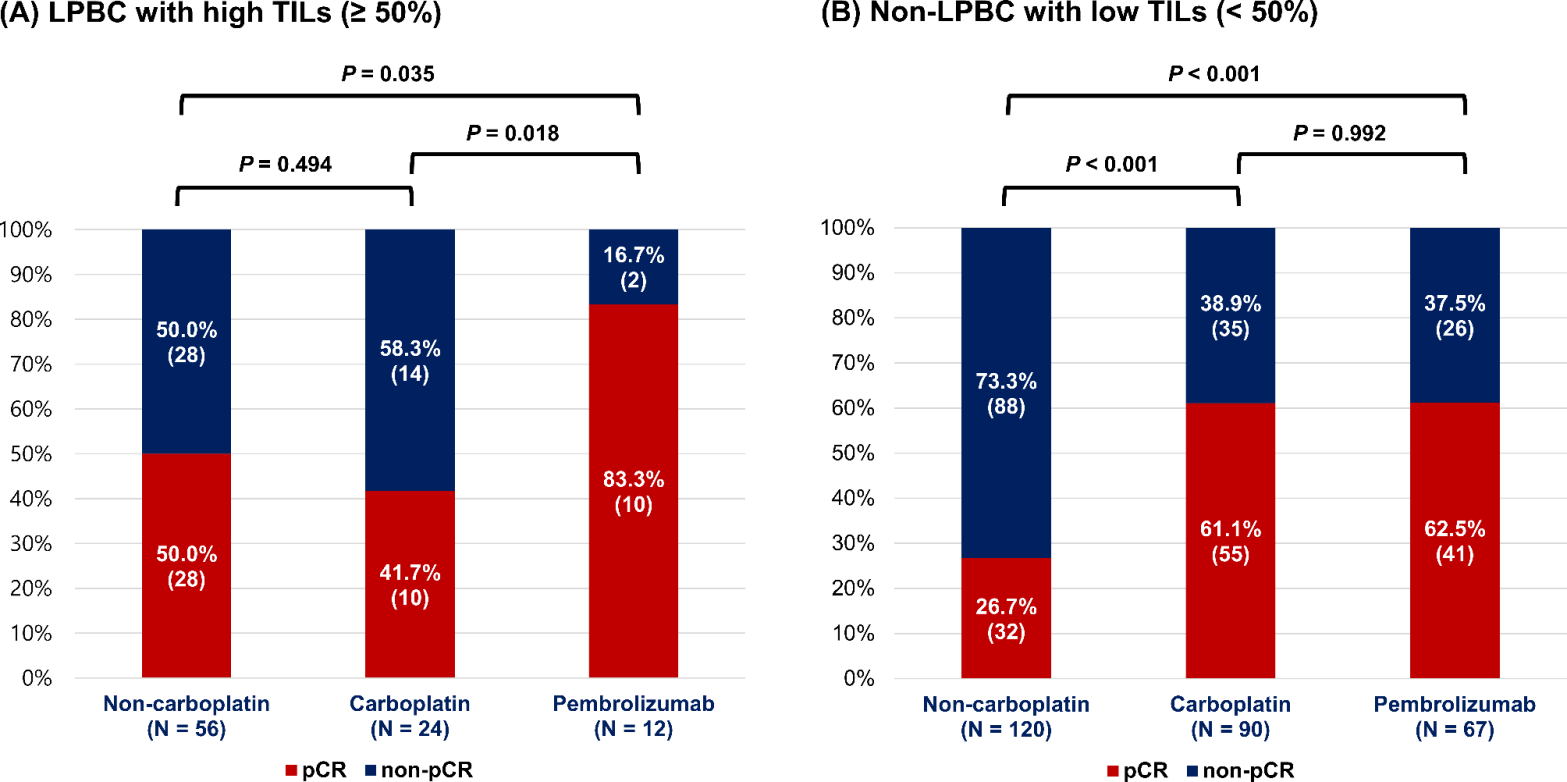
**Pathologic complete response (pCR) rate according to neoadjuvant treatment regimens stratified by stromal tumor-infiltrating lymphocytes (sTILs)**: (**A**) in lymphocyte-predominant breast cancer (LPBC) with high sTILs (≥ 50%), and (**B**) in non-LPBC with low sTILs (< 50%)

In the cohort with available sTILs data, 24.9% (92/369) were classified as LPBC (sTILs ≥ 50%), and 75.1% (277/369) were classified as non-LPBC (sTILs < 50%). Within the patients with LPBC, the pCR rates for the non-carboplatin- (28/56 [50.0%]) and carboplatin- (10/24 [41.7%]) chemotherapy groups were similar, whereas the pCR rate for the pembrolizumab-chemotherapy group (10/12 [83.3%]) was significantly higher compared to the other two groups (Fig. 2A). In the multivariable analysis (Table 3), the pembrolizumab-chemotherapy group (adjusted OR, 6.40; 95% CI, 1.09–37.47; *P* = 0.040) showed a significant increase in the pCR rate compared to those in the non-carboplatin group, whereas the carboplatin group showed no difference from the non-carboplatin group (adjusted OR, 1.01; 95% CI, 0.31–3.26; *P* = 0.986).

**Table 3:** Odds ratio (OR) and 95% confidence interval (CI) of treatment regimen for pathologic complete response stratified by stromal tumor-infiltrating lymphocytes

Cohort		Regimen	Multivariable analysis		
			adjusted OR^*^	95% CI	*P*
All patients with sTILs	LPBC	Non-carboplatin	Ref.		
		Carboplatin	1.01	0.31–3.26	0.986
		Pembrolizumab	6.40	1.09–37.47	0.040
	Non-LPBC	Non-carboplatin	Ref.		
		Carboplatin	4.09	2.24–7.48	< 0.001
		Pembrolizumab	4.47	2.30–8.67	< 0.001
Cohort		Regimen	Multivariable analysis		
			adjusted OR^†^	95% CI	*P*
cN + patients with sTILs	LPBC	Non-carboplatin	Ref.		
		Carboplatin	0.98	0.30–3.23	0.977
		Pembrolizumab	11.45	1.17-112.43	0.036
	Non-LPBC	Non-carboplatin	Ref.		
		Carboplatin	4.36	2.17–8.78	< 0.001
		Pembrolizumab	3.94	1.91–8.13	< 0.001

^*^Adjusted for age (≤ 50 vs. > 50), histologic grade (1 vs. 2 vs. 3), clinical tumor stage (1 vs. 2 vs. 3 vs. 4), and clinical nodal stage (0 vs. 1 vs. 2 vs. 3). ^†^Adjusted for age (≤ 50 vs. > 50), histologic grade (1 vs. 2 vs. 3), clinical tumor stage (1 vs. 2 vs. 3 vs. 4), and clinical nodal stage (1 vs. 2 vs. 3). sTILs, stromal tumor-infiltrating lymphocytes; LPBC, lymphocyte-predominant breast cancer. Supplemental Table 1. Baseline characteristics of patients according to neoadjuvant chemotherapy regimen with baseline sTIL values (*n* = 369)

Among the patients with non-LPBC, the non-carboplatin-chemotherapy group (32/120 [26.7%]) had a significantly lower pCR rate compared to the other groups, while the carboplatin- (55/90 [61.1%]) and pembrolizumab- (41 of 67 [62.5%]) chemotherapy groups had similar pCR rates (Fig. 2B). The multivariable analysis showed that both the carboplatin (adjusted OR, 4.36; 95% CI, 2.17–8.78; *p* < 0.001) and pembrolizumab (adjusted OR, 3.94; 95% CI, 1.91–8.13; *p* < 0.001) groups significantly increased pCR rate compared the non-carboplatin group (Table 3).

Consistent outcomes were noted in the subset of 295 clinically node-positive disease. Among LPBC patients, the pembrolizumab-chemotherapy group demonstrated a significantly higher pCR rate (9/10 [90.0%]) than the other groups. In non-LPBC patients, the non-carboplatin-chemotherapy group exhibited a lower pCR rate compared to the carboplatin- and pembrolizumab-chemotherapy groups (Supplementary Fig. 2). The multivariable analysis confirmed these findings, indicating that the pembrolizumab-chemotherapy regimen was associated with a higher likelihood of pCR in LPBC compared to the other two groups (Table 3). However, the probability of pCR did not differ between the carboplatin- and pembrolizumab-chemotherapy groups in non-LPBC.

**Fig. 3:**
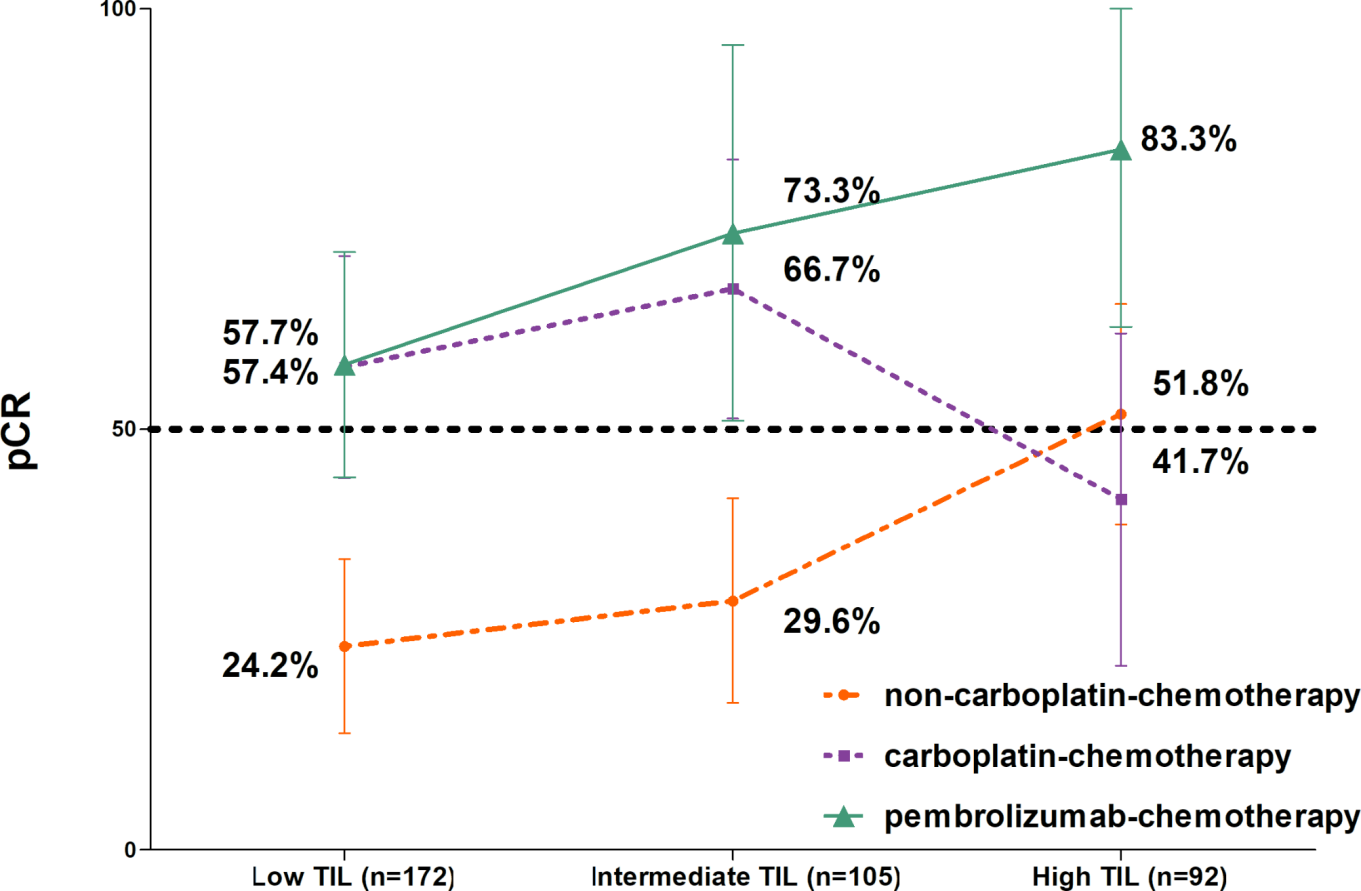
Changes in pathologic complete response (pCR) rate according to stromal tumor-infiltrating lymphocytes (sTILs) level in each neoadjuvant treatment regimen

Lastly, we divided the non-LPBC group into two subgroups based on TIL levels (low [sTIL ≤ 10%] and intermediate [sTIL, 11–49%]) and compared the pCR rates of each regimen among the three groups defined by sTILs. In this analysis, the pCR rate for the pembrolizumab-chemotherapy group showed a consistent increase across the TILs subgroups: 57.7% in the low-, 73.3% in the intermediate-, and 83.3% in the high (Fig. 3). However, the pCR rates for the carboplatin-chemotherapy group were 57.4% and 66.7% in the low and intermediate groups, respectively, but dropped to 41.7% in the high group. The pCR rates for the non-carboplatin chemotherapy group were low at 24.2% and 29.6% in the low and intermediate groups, respectively, but increased to 51.8% in the high group.

## Discussion

While our real-world data represent the early experiences following the introduction of pembrolizumab, we observed a numerical increase in pCR rates across different regimens, consistent with the results of the KeyNote-522 study: non-carboplatin-chemotherapy, 32.4%; carboplatin- chemotherapy, 57.5%; pembrolizumab- chemotherapy, 67.8%. Furthermore, in TNBC cases with high sTILs, referred as to LPBC, the addition of pembrolizumab to chemotherapy resulted in a pCR rate exceeding 80%, whereas the incorporation of carboplatin into AC/T chemotherapy did not enhance the response. Conversely, in non-LPBC, the inclusion of carboplatin in AC/T significantly enhanced the pCR rate. However, the introduction of pembrolizumab did not demonstrate additional efficacy in this subgroup.

When the patients were classified into three groups by sTIL level (Fig. 3), the pembrolizumab-chemotherapy group correlated with increased pCR rates in higher TILs subgroups, whereas carboplatin-chemotherapy group did not show this positive trend. In addition, the non-carboplatin-chemotherapy group exhibited a trend of rising pCR rates corresponding to increasing TILs; however, these rates remained noticeably lower than those observed in the pembrolizumab-chemotherapy group. The analysis demonstrates that pembrolizumab-chemotherapy is particularly effective, and its efficacy appears to be related to sTIL levels, suggesting a potential immunological synergy. As supporting evidence, the KEYNOTE-086 trial showed that linear with increasing sTILs is related to the efficacy of pembrolizumab monotherapy in advanced TNBC [22].

It is well established that TNBC tumors exhibit a tumor microenvironment (TME) enriched with immune cells, characterized by high levels of sTILs, PD-L1-positivity and up-regulated immune gene signatures [16, 17, 23, 24]. These immune biomarkers exhibit correlations [16, 24]. Presently, in advanced TNBC, pembrolizumab is exclusively indicated for patients with high PD-L1 expression, as identified by the 22C3 pharmDx assay with a cutoff of a combined positive score (CPS) of 10 [25, 26]. However, in early TNBC, pembrolizumab is administered to all patients regardless of PD-L1 status [1, 2]. Nevertheless, pembrolizumab-based immunochemotherapy achieved a pCR rate of 68.9% in the PD-L1-positive group compared to 45.3% in the PD-L1-negative group [1]. Considering positive correlation between PD-L1 status and TILs level [24, 27], these findings suggest that pembrolizumab-based immunochemotherapy may enhance response in TNBC with a more immunogenic TME.

Although in the KEYNOTE-522 trial, the application of pembrolizumab is recommended for all patients with stage II-III TNBC regardless of immune status [1], our findings suggest that the application of pembrolizumab should be more actively considered in patients with high sTILs within this population. Furthermore, among non-LPBC patients, those with non-pCR remain at high risk, irrespective of the neoadjuvant chemotherapy regimen used. The application of novel antibody-drug conjugates or sequential or combination therapies with currently available agents such as capecitabine, pembrolizumab, or olaparib can be considered for these high-risk patients [28].

The pronounced effect of pembrolizumab in LPBC suggests several implications for treatment de-escalation strategies. First, it highlights the potential feasibility of omitting anthracyclines or reducing the duration of chemotherapy in neoadjuvant regimens that incorporate pembrolizumab for LPBC. In the Neo-PACT trial, which evaluated the efficacy of an anthracycline-free neoadjuvant regimen of carboplatin and docetaxel combined with pembrolizumab in early TNBC, high sTIL as biomarkers for the degree of immune enrichment was predictive of pCR [16]. Additionally, these findings support that the efficacy of pembrolizumab in TNBC is positively correlated with sTIL levels and may reach its maximum in cases classified as LPBC [20, 21, 29], as observed in our study. Ongoing trials are exploring immunochemotherapy with de-escalation of backbone chemotherapy, such as anthracyclines-free regimen or 4 cycles taxane-platinum [30, 31], based on sTIL levels.

A second consideration is the potential for de-escalation of adjuvant pembrolizumab. Based on the design of the KEYNOTE-522 trial, current guidelines recommend continuation of pembrolizumab in the adjuvant setting regardless of treatment response or immune-related biomarkers. However, the updated analysis from the KEYNOTE-522 trial demonstrated no differences in EFS or overall survival between treatment arms in patients who achieved pCR [9, 10]. Moreover, pooled data from two studies revealed excellent survival outcomes in patients with high sTILs who achieved pCR following anthracycline-free neoadjuvant chemotherapy [32]. In line with this, for stage I TNBC with high sTIL levels, omission of adjuvant chemotherapy has been proposed due to the low recurrence risk [33, 34]. These findings collectively suggest that adjuvant pembrolizumab de-escalation based on sTIL levels may be a promising approach [28], warranting further investigation.

Intriguingly, we noted that carboplatin increased pCR rate in non-LPBC (sTIL < 50%). While high sTIL levels were associated with pCR in neoadjuvant chemotherapy trials with carboplatin in TNBC, it remains unknown whether carboplatin addition can elevate pCR in non-LPBC.

Among previous trials, the GeparSixto study demonstrated a pCR rate of 74% with carboplatin compared to 43% without carboplatin in LPBC (sTIL ≥ 60%), while in non-LPBC cases, the rates were 46% with carboplatin and 34% without carboplatin, respectively [4]. In contrast, the study by Dieci MV et al. showed that carboplatin significantly increased the pCR rate in non-LPBC (sTIL < 60%) [35]. The discrepancy between the two studies may be partly explained by differences in backbone chemotherapy regimens; the GeparSixto study utilized weekly paclitaxel and liposomal doxorubicin for 18 weeks, whereas the study by Dieci MV et al. employed AC followed by weekly paclitaxel for 24 weeks. Further investigations are necessary to fully understand the distinct response patterns to incorporation of carboplatin based on TIL levels in TNBC. Additionally, ongoing trials could assess the potential of novel agents, such as TROP2-targeting antibody-drug conjugates, to enhance response rates in non-LPBC cases [36].

Our study has several limitations. First, our study represents early-phase real-world outcomes following the implementation of pembrolizumab-based immunochemotherapy for early-stage TNBC, which has led to a relatively small cohort in the pembrolizumab group, with an even smaller subset of high sTILs cases. Second, survival outcomes were not analyzed in this study, primarily because patients treated with pembrolizumab received the therapy relatively recently, with a median follow-up of only 9 months. Third, valuable clinical factors such as the PD-L1 CPS and residual cancer burden (RCB) class were not evaluated in relation to sTILs, as these indicators were not assessed in tumor samples collected a decade ago. A comprehensive analysis exploring the impact of various immune-related biomarkers on clinical outcomes, including pCR, RCB, and survival, will be necessary in future studies.

Concerns have been raised regarding intraobserver and interobserver heterogeneity in the assessment of sTILs. To address these challenges, an online educational resource (www.tilsinbreastcancer.org/pitfalls) has been developed [37], and efforts to implement automated assessment have been initiated [38]. Nevertheless, in our study, sTILs were evaluated by a specialized pathologist with eight years of experience in sTIL assessment. Furthermore, a previous study demonstrated acceptable agreement in TIL scoring when applying the currently proposed guidelines [19, 39].

Despite these limitations, our study leveraged sTILs and addressed differential responses across three different regimens: carboplatin-based and non-carboplatin-based, as well as pembrolizumab immunochemotherapy. Our findings may provide a foundation for tailoring immunochemotherapy based on stromal TILs in TNBC.

In conclusion, our real-world data consistently demonstrates an increased pCR rate with the addition of carboplatin and pembrolizumab. In LPBC, the addition of carboplatin did not result in an elevated pCR rate. However, the addition of pembrolizumab tended to boost the pCR rate, surpassing 80%. On the other hand, among patients with non-LPBC, the addition of carboplatin significantly increased the pCR rate, while the addition of pembrolizumab did not have the same effect. Further efforts should be made to improve the response to pembrolizumab-chemotherapy for patients with low baseline sTIL levels.

## Electronic Supplementary Material

Below is the link to the electronic supplementary material.

Supplementary Material 1

## Data Availability

Ahn SG and Cha YJ, co-corresponding authors, had full access to all the data in the study and takes responsibility for the integrity of the data and the accuracy of the data analysis. The data that support the findings of this study are available from the corresponding author, upon reasonable request.
