# Supplementary material for: Stromal tumor-infiltrating lymphocytes and pathologic response to neoadjuvant chemotherapy with the addition of platinum and pembrolizumab in TNBC: a single-center real-world study
Source: Breast Cancer Res. 2024 Dec 18;26:182. doi: 10.1186/s13058-024-01944-0 (PMC11657785; doi:10.1186/s13058-024-01944-0)
Supplement: Supplementary file 1 — Supplementary Material 1 [file 13058_2024_1944_MOESM1_ESM.docx]

**Supplemental Table 1. Baseline characteristics of patients according to neoadjuvant chemotherapy regimen with baseline sTIL values (n = 369).**

|  | Non-carboplatin (N = 176) | Carboplatin (N = 114) | Pembrolizumab (N = 79) | Total (N =369) | *P* |
| --- | --- | --- | --- | --- | --- |
| Age, n (%) |  |  |  |  | 0.170 |
| ≤ 50 | 93 (52.8) | 73 (64.0) | 45 (57.0) | 211 (57.2) |  |
| > 50 | 83 (47.2) | 41 (36.0) | 33 (43.0) | 158 (42.8) |  |
| Germline BRCA1/2 mutation^*^ |  |  |  |  | 0.064 |
| Yes | 68 (84.0) | 63 (70.0) | 53 (81.5) | 184 (78.0) |  |
| No | 13 (16.0) | 27 (30.0) | 12 (18.5) | 52 (22.0) |  |
| Clinical tumor stage |  |  |  |  | 0.781 |
| 1 | 13 (7.4) | 6 (5.3) | 6 (7.6) | 25 (6.8) |  |
| 2 | 123 (69.9) | 86 (75.4) | 59 (74.7) | 268 (72.6) |  |
| 3 | 31 (17.6) | 18 (15.8) | 13 (16.5) | 62 (16.8) |  |
| 4 | 9 (5.1) | 4 (3.5) | 1 (1.3) | 14 (3.8) |  |
| Clinical nodal stage |  |  |  |  | 0.280 |
| 0 | 41 (23.3) | 23 (20.2) | 10 (12.7) | 74 (20.1) |  |
| 1 | 65 (36.9) | 50 (43.9) | 42 (53.2) | 157 (42.5) |  |
| 2 | 58 (33.0) | 34 (29.8) | 19 (24.1) | 111 (30.1) |  |
| 3 | 12 (6.8) | 7 (6.1) | 8 (10.1) | 27 (7.3) |  |
| Clinical Stage, n (%) |  |  |  |  | 0.296 |
| I,IIA | 43 (24.4) | 22 (19.3) | 12 (15.2) | 77 (20.9) |  |
| IIB | 53 (30.1) | 40 (35.1) | 36 (45.6) | 129 (35.0) |  |
| IIIA | 61 (34.7) | 41 (36.0) | 22 (27.8) | 124 (33.6) |  |
| IIIB | 7 (4.0) | 4 (3.5) | 1 (1.3) | 12 (3.3) |  |
| IIIC | 12 (6.8) | 7 (6.1) | 8 (10.1) | 27 (7.3) |  |
| Histologic grade^*^, n (%) |  |  |  |  | 0.385^†^ |
| 1 | 4 (2.3) | 0 | 1 (1.3) | 5 (1.4) |  |
| 2 | 81 (46.0) | 53 (46.9) | 43 (54.4) | 177 (48.1) |  |
| 3 | 91 (51.7) | 60 (53.1) | 35 (44.3) | 186 (50.5) |  |
| sTILs, median (interquartile range), % | 20 (10-60) | 20 (3-40) | 10 (3-25) | 20 (3-40) | <0.001 |
| sTILs^*^, % |  |  |  |  | 0.009 |
| <50 | 120 (68.2) | 90 (78.9) | 67 (84.8) | 277 (75.1) |  |
| ≥50 | 56 (31.8) | 24 (21.1) | 12 (15.2) | 92 (24.9) |  |
| Surgery type |  |  |  |  | 0.967 |
| Lumpectomy | 109 (61.9) | 69 (60.5) | 48 (60.8) | 226 (61.2) |  |
| Mastectomy | 67 (38.1) | 45 (39.5) | 31 (39.2) | 143 (38.8) |  |

^*^Missing values

^†^*P*-values were obtained with the Fisher’s exact test.

HG, histologic grade; SD, standard deviation; TILs, tumor-infiltrating lymphocytes


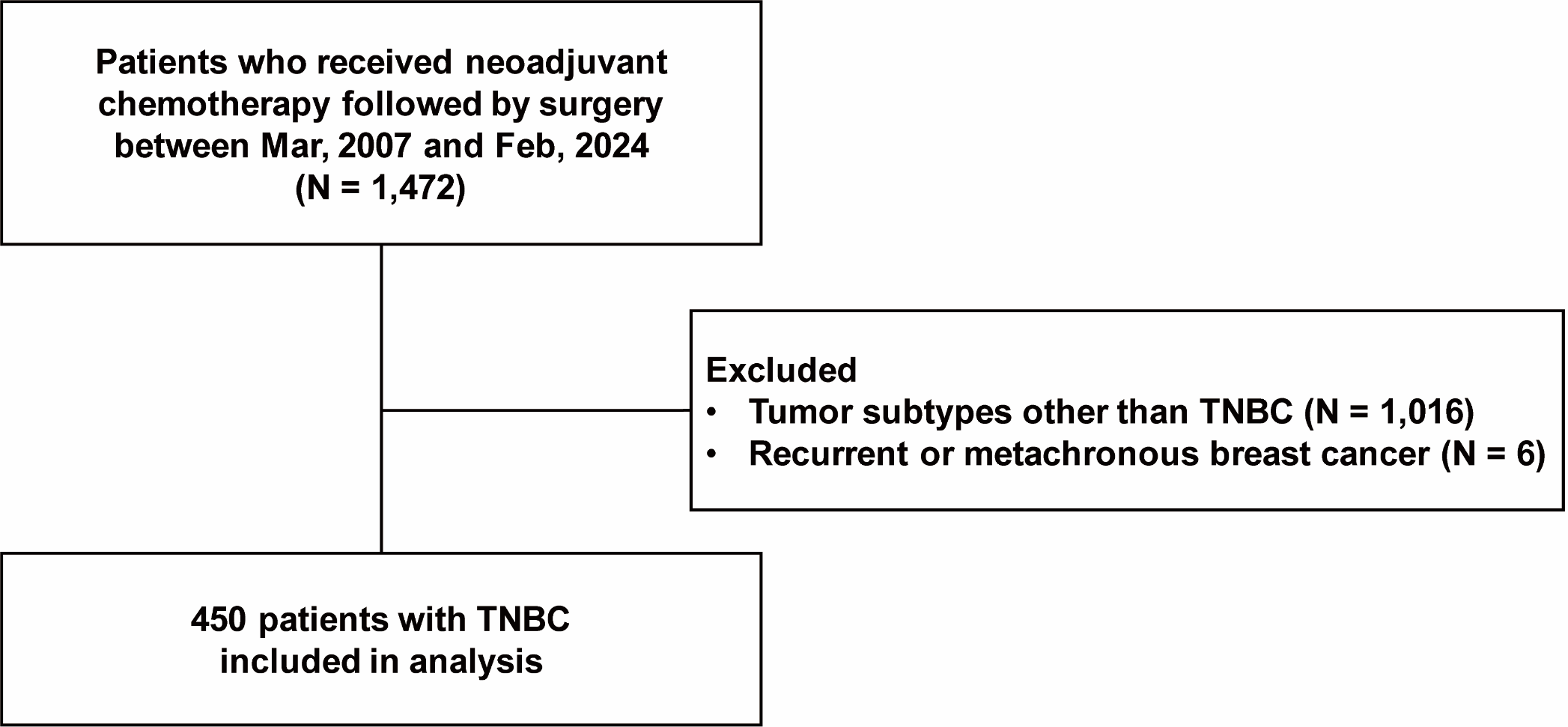


Figure 1.


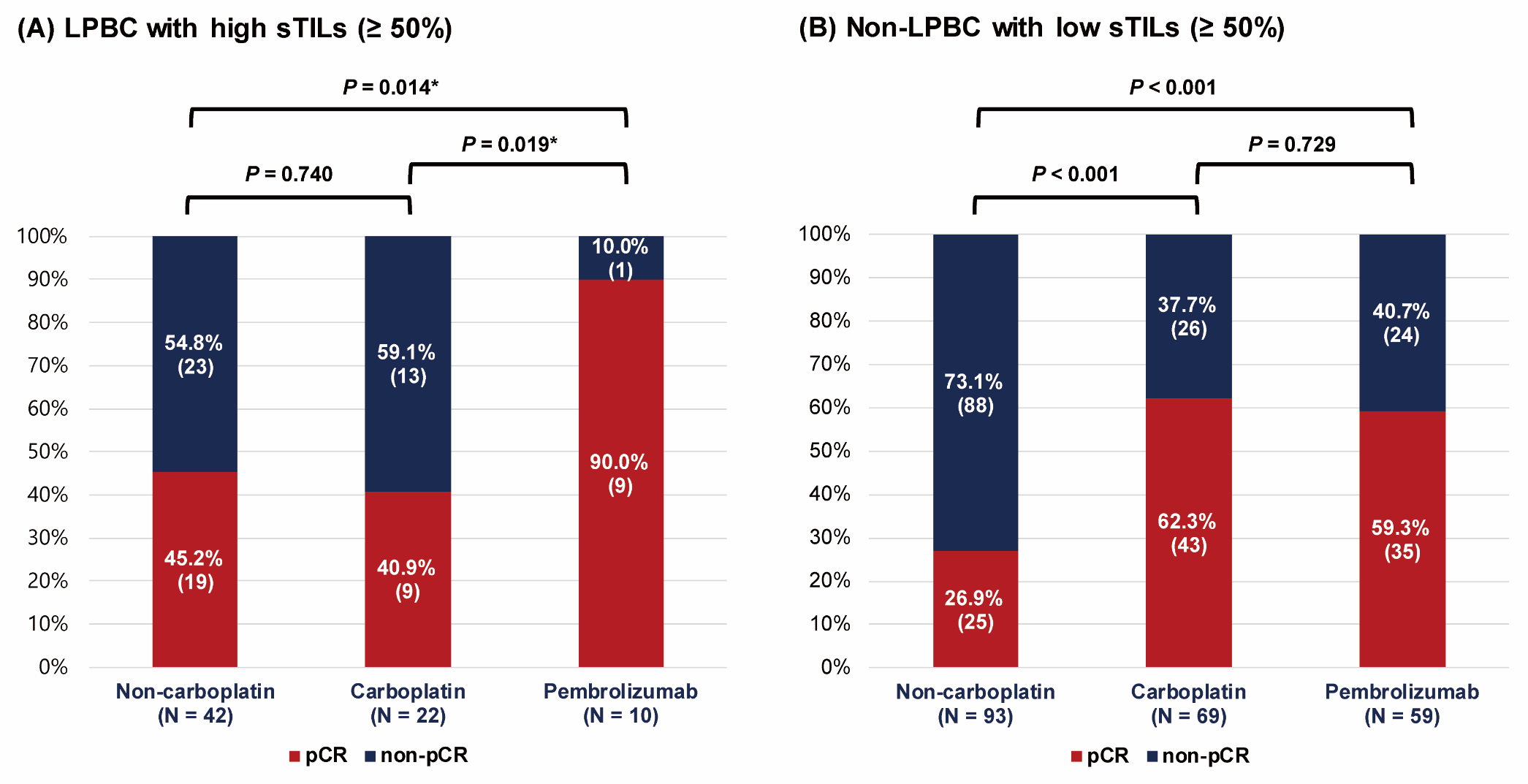


Figure 2.
